# Twelve Tips for Medical Education Leadership to Help Faculty in the Transition to Online Teaching

**DOI:** 10.15694/mep.2020.000275.1

**Published:** 2020-12-09

**Authors:** Marie Norman, Eleanor Sharp, Carla Spagnoletti, Benjamin Miller

**Affiliations:** 1 Institute for Clinical Research Education; 2 Paul C. Gaffney Division of Pediatric Hospital Medicine; 3 Division of General Internal Medicine

**Keywords:** medical education, online learning, COVID-19, virtual education, leadership

## Abstract

This article was migrated. The article was marked as recommended.

The COVID-19 pandemic has had innumerable profound impacts, many of which have resulted in irrevocable changes to both the practice of medicine and the teaching of medicine. Medical educators have adapted to the need for social distancing by providing virtual educational opportunities for their learners, often without training or experience in online teaching. Because these educators require both professional development and technical support for their online teaching to be sustainable and effective, departments need to provide immediate help and support. These twelve tips are designed for those in leadership roles who have recognized the need for investment in online education and are interested in strategies to ensure the success of their educational programs.

## Introduction

In response to the COVID-19 pandemic, medical educators around the world have suddenly found themselves moving educational activities online ( Bao, 2020; Wolanskyj-Spinner, 2020), from virtual rounding ( Dedeilia *et al.*, 2020; Hofmann *et al.*, 2020) to morning report and case discussions ( Kondziolka, Couldwell and Rutka, 2020), to journal clubs ( Codispoti *et al.*, 2020; Tomlinson, Hendricks and Cohen-Gadol, 2020) and even full-length courses ( Ferrel and Ryan, 2020). They have often moved rapidly, with little preparation, and minimal institutional support ( Anderson, 2020; Kamenetz, 2020). Medical educators have embraced the charge bravely, rising to the challenge of transitioning their teaching content and interaction with their learners online while also adapting to unfamiliar formats. However, they are often underprepared, as many faculty members lack formal training in the delivery of online courses, and many more may struggle with the technology required to get connected *.*

Recent articles have described the effects of this shift on medical education ( Parisi *et al.*, 2020; Rose, 2020; Sandhu and de Wolf, 2020; Wayne, Green and Neilson, 2020; Weiner, 2020), discussed student and faculty responses to online learning ( Agarwal and Kaushik, 2020; Muflih *et al.*, 2020), provided case studies of specific teaching approaches (Chick *et al.*, 2020; Parisi *et al.*, 2020; Zhou *et al.*, 2020), and offered tips for faculty making this transition ( Gewin, 2020; Reyna, 2020). However, little advice has been delineated for institutional leadership and departments to support their teaching faculty under these challenging circumstances.

Many administrators are eager to support their educators but are unsure about how or where to start. Thus, we wrote the following twelve tips to provide tangible and actionable ways for department leadership to assist faculty as they transition to online education. Given the urgent and unexpected nature of the transition to the virtual space, the majority of these tips are designed to address immediate needs. However, the consequences of the pandemic and the shift to online education are likely to be long-lasting ( Branswell, 2020; Razavi, 2020; Rose, 2020). Thus, the last tip provides advice on building institutional capacity in anticipation of a sustained investment in online education.

## 1. Ensure faculty have the necessary hardware

A recent report shows that large percentages of faculty do not have access to an institutionally provided laptop (45%) or desktop (40%) ( Brooks and Grajek, 2020). Many institutions expect that faculty will use their own computers for online instruction. However, their personal devices may be outdated or not configured to run the latest versions of the applications required for online teaching. Faculty may be connecting from areas without reliable internet access ( McMurtrie, 2020), which can result in inconsistent streaming. Moreover, faculty may have to share these personal computers with other family members working from home. As a result, there can be gaps “between the work faculty are being asked to do post-COVID and the tools that are being provided to conduct that work” ( Brooks and Grajek, 2020). To address and mitigate these gaps, survey your faculty members to determine what hardware (i.e., laptop, desktop, tablet) and equipment (i.e., headset, microphone, webcam) they have and what technology they still need. Ask about the status of their internet connectivity. Make sure faculty know what tools they will need to teach online and be prepared to fill any gaps.

## 2. Familiarize faculty with their Learning Management System

Faculty at all major universities have access to a learning management system (LMS), for example Canvas (Instructure, Salt Lake City UT), Blackboard (Blackboard, Inc, Washington DC), or Moodle (Moodle Pty Ltd, West Perth, Australia). Yet some faculty (particularly clinical faculty) may not know that these platforms exist. Others may be unfamiliar with the full capabilities of the LMS, particularly those functions that promote interaction in online teaching sessions (e.g., discussion boards, wikis, plug-ins to tools such as VoiceThread (VoiceThread LLC, Boca Raton FL) ( Brooks and Grajek, 2020). These skill gaps limit their ability to teach effectively online. Thus, one of the simplest things departments can do to help faculty get up to speed online is to offer LMS training, focusing both on basic features (e.g., how to add, delete and edit content, how to use the gradebook) and on the features that will inspire the most student engagement. This training can often be provided by the university teaching center, if your university has one, or by an experienced staff or faculty member from your department. In a pinch, you can assemble links to resources, such as training videos and technical support, provided by the LMS company itself. Making sure that faculty are facile with the LMS and its tools is likely to instill confidence while also alerting them to forms of online content and interaction they might not otherwise have known. Over time, departments can expose faculty to a wider array of tools to integrate into their courses.

## 3. Provide exemplars

Many faculty members have never taken an online course, let alone taught online. Thus, it may be difficult to imagine what online teaching looks like, or to set appropriate expectations ( Schmidt, Tschida, Christina and Hodge, 2016). It is important for faculty to see examples of online courses and modules before they work on their own. There are many examples online; however, they often involve subject matter (e.g., meditation, yoga, guitar) that can be difficult for faculty in academic medicine to relate to their own teaching practice. They may also represent a different type of online offering altogether (e.g., a self-paced video-based module rather than an instructor-led module with tasks and assignments) thus creating confusion about what, precisely, the final product should be. To find the right kind of model, consider asking your institution’s teaching center to identify and provide guest access to well-designed courses that are similar to those your faculty teach. Request courses that represent like content (e.g., physical exam or procedural skills, research design, medical communication), teaching methods (e.g., problem solving, case-based discussion), student level (e.g., medical student, resident, fellow), and class size. The closer the model is to your own faculty members’ experience, the more likely it will speak to them. If possible, provide a range of models, not just a single example, to illustrate different options and approaches. Consider asking the instructors who developed these courses to attend a faculty meeting or professional development event to talk about the course design process and/or the teaching experience.

## 4. Arrange for an assistant to support virtual class sessions

As sessions move online, synchronous teaching increasingly takes place in video conferencing platforms such as Zoom (Zoom Video Communications Inc., San Jose, CA), Microsoft Teams (Microsoft Corporation, Redmond, WA), Cisco Webex (Cisco Systems, Milpitas, California), and GoToMeeting (LogMeIn Inc., Boston, MA). These tools create opportunities (for example, the ability for students to participate even when not in the same physical location, the seamless integration of polling, and easy screen sharing capabilities) but also challenges. Instructors must manage new technologies and navigate an unfamiliar set of logistics while also adjusting to a different kind of pedagogy. To help faculty manage the added cognitive burden, consider providing the services of a staff member or teaching assistant who can sit in on virtual class sessions to help monitor the chat window, resolve technical problems for students, moderate breakout rooms, and assist if the instructor encounters challenges. Knowing they have help with technical and logistical issues allows them to focus their energy and creativity on teaching.

## 5. Consolidate technology tools

Choice is good, but too many choices can be paralyzing. If faculty are relatively new to online teaching, it may be wise not to provide too many choices of tools and technologies. Instead, recommend a small set that come highly recommended and have been well-vetted by people in your field. Make it clear to faculty what each of these tools is for (e.g., housing course materials, hosting synchronous class sessions) and provide robust training and support resources for each. Consider using the same tools for other departmental purposes so that faculty get comfortable using them. For example, you might use Zoom for virtual department meetings while recommending Zoom for teaching as well. Using a limited number of good tools reduces the cognitive burden for faculty while also creating a more consistent and manageable learning experience for students, who must otherwise master different platforms and tools for different training purposes. As faculty become more adept at online teaching, departments can consider purchasing site licenses for additional tools targeting more sophisticated uses, such as the development of interactive video content or enabling media-rich discussions.

## 6. Use templates and rubrics

Faculty are accustomed to having latitude in how they organize and run their courses, an important facet of academic freedom. However, as instructors are learning how to design and teach online courses, structure and guidance in this context may be appreciated. Departments should consider providing templates and rubrics to guide online course creation. Templates help faculty identify key course components (e.g., course overview, course objectives, modules, synchronous activities, asynchronous activities) as well as the logical steps in course design ( Borgemenke, Holt and Fish, 2013). Rubrics, on the other hand, designate the marks of quality in an online course. Two of the most well-known rubrics are the Quality Matters Rubric ( *QM Higher Education Rubric,* 2018) and the Online Learning Consortium’s Quality Score Card ( *OLC Quality Scorecard*, 2018). Templates and rubrics help to develop a base level of standardization across courses, which leads to a shared vocabulary among faculty, while ensuring a more consistent, high-quality experience for learners.

## 7. Appoint a staff member to oversee course consistency

Most students have taken in-person courses since their elementary school days. They understand what classrooms look like and how these courses generally operate. Online, however, that familiar structure is gone, and students can easily feel disoriented and confused. Dykman and Davis (2018) recommend that all courses in a program have a similar look and feel to make courses easier for students to navigate. To achieve this, it can be beneficial for departments to assign a staff member the job of reviewing courses to ensure consistency. For instance, do all courses have clearly articulated learning objectives? Do they use terminology (e.g., module, session, unit) in consistent ways? Do syllabi reside in a consistent location? Ideally, staff members in this role would have a background in instructional design, but that is not strictly necessary, as long as they are armed with the templates and rubrics described above.

## 8. Supply relevant resources

Teaching online entails a specific set of skills for which few medical educators have had explicit training. Until such training can be provided, consider buying your teaching faculty a small set of helpful books about online teaching. A gift is always appreciated, and this gesture may help to foster good will among faculty who may be feeling overwhelmed. Books provide basic information and advice about designing and teaching online courses which most faculty need. Moreover, the best books ground their advice in research-based learning principles ( Ambrose *et al.*, 2010; Brown, Roediger and McDaniel, 2014) which are a necessary foundation for teachers working in any modality. Finally, a shared set of books helps to foster a vocabulary among the faculty, making communication more streamlined and effective. Books we recommend include: *Small Teaching Online: Applying Learning Science to Online Classes* by Darby and Lang (2019) and *The Online Teaching Survival Guide* by Boettcher and Conrad (2016).

## 9. Capitalize on existing professional development

Copious research points to the necessity of providing robust professional development to faculty new to teaching online (Shelton *et al.,* 2014; Vaill and Testori, 2012; Mohr and Shelton, 2017; Hampton *et al.*, 2020). Your medical school may have its own teaching center or faculty development unit. If so, explore whether it offers workshops and trainings on online teaching for your faculty. If not, your university’s teaching center may be able to provide programming on online course design and teaching (independent of the platform-specific training mentioned above in #2), customized with examples from your field. If these are not options, your department may be able to purchase a group subscription to a seminar series focused on online teaching (e.g., Magna Online Seminar Series, Magna Publications, Madison, WI) or simply assemble a set of useful resources from professional organizations like the Online Learning Consortium (Online Learning Consortium, Boston MA) and Educause (Educause, Louisville, CO): focused on online and technology-enhanced education. Consider asking a graduate student to assemble a set of recommended sources.

## 10. Create a forum for faculty to share tips and ideas

Faculty often learn best from one another. Thus, it can be helpful to provide a forum for faculty to compare notes, share ideas, and learn from one another. Forums such as these already exist; for instance, a number of national Facebook groups were created after institutions moved online in response to COVID-19. One called “Pandemic Pedagogy” seems to have gained considerable momentum ( *Pandemic Pedagogy for Visual Arts Professors*, 2020), splitting into discipline-specific subgroups. There is even one group called “STEM Faculty Blundering Through Remote Teaching in a Pandemic” ( *STEM faculty blundering through remote teaching in a pandemic*, 2020)! However, because national groups on social media can be large and overwhelming, it may best to see if there is an existing group at your institution or to create (or encourage faculty to create) a forum of their own using social media and collaboration tools, e.g., Slack (Slack Technologies, San Francisco CA) or Microsoft Teams (Microsoft Inc, Redmond WA).

## 11. Hire a student or staff member to organize a repository of online content

Many faculty developing online courses believe that they must record all their lectures as videos, a daunting task in the best of times ( Norman, 2017). However, there are other options, including using materials that already exists in the public sphere: TEDMed talks, podcasts, NIH-produced video, even whole curricula. Open Educational Resources Commons ( *OER Commons*, 2020), for example, provides free educational materials from a wide range of subjects and learner levels. While busy faculty may not have the time to search for these materials, it is a perfect job for a staff member or graduate student in your department - someone familiar with the types of courses your faculty teach and knowledgeable enough to evaluate the quality of the content. Hiring such a person to find, organize, and catalog a set of department-relevant multimedia materials can be a wise investment. To make sure the repository they assemble is as useful as possible to faculty, ensure that the materials are systematically organized and indexed by topic, source, length, and content type (e.g., video, podcast, curriculum), and include a brief description.

## 12. Invest in staffing and resources to provide sustained support

In anticipation of a world where online and hybrid medical education are increasingly common, there is an undisputed need to offer robust, long-term support for faculty teaching online, and thus the need to build the capacity necessary to do so. There are multiple layers involved. First, a mechanism must be in place to provide ongoing faculty development, preferably within your own School of Medicine or School of Health Sciences. Second, you should consider hiring an instructional designer. Instructional designers (IDs) are trained to work closely with faculty to develop instructional materials, often enlisting technology tools. A good ID combines pedagogical know-how and keen design aesthetic with technical expertise, making him or her a valuable asset to a department. Third, consider hiring a video producer to record and edit video and audio to create high-quality multimedia materials. Finally, consider building a video and audio recording studio. This does not have to be an expensive venture. For example, Pennsylvania State University pioneered the “One Button Studio,” designed to allow faculty to record lecture video themselves, but with professional lighting and backdrops ( *Welcome to the One Button Studio*, 2020). It may also be possible to share studio facilities between several departments and programs. While the aforementioned steps are more involved and require an investment of time and financial support, they have the potential to create lasting and impactful opportunities for medical educators to thrive in the online space.

## Conclusion

Though the rapid transition to online teaching has been sparked by the novel coronavirus pandemic, it is likely that online and hybrid education will become mainstays in medical education in the future. We hope these tips can assist institutions to manage this transition with greater purpose and focus. We recognize that many of these aforementioned tips require an investment (some financial, most with at least time and energy), which may seem daunting at first. It would be reasonable to approach this list in a stepwise fashion, allocating resources to the earlier tips first before tackling the later ones. Both the quantity and quality of online teaching may become a discerning factor for students applying to medical education programs. Thus, those programs that are proactive in their approach could attract higher quality matriculants and expect to produce higher quality graduates. In the end, we anticipate that institutional leaders who make progress through these suggestions may experience a competitive advantage in the medical education marketplace for years to come.

## Take Home Messages

• The consequences of the COVID19 pandemic are likely to be long-lasting, underscoring the importance of sustainable online learning experiences for educators and learners.
• Medical educators are in need of professional development opportunities and institutional technical support to assist in this period of transition.
• By leveraging existing resources and capitalizing on the benefits of technology, departmental leadership can provide assistance to educators as they adapt their pedagogy to the online space.
• Timely investments to optimize the online learning experience will have lasting effects for both educators and learners alike.

## Notes On Contributors

**Marie Norman** is co-author of How Learning Works: Seven Research-Based Principles for Smart Teaching. She directs the IDEA Lab at the University of Pittsburgh’s Institute for Clinical Research Education. ORCiD: https://orcid.org/0000-0003-1307-0115

**Eleanor Sharp** is Pediatric Hospital Medicine fellow at UPMC Children’s Hospital of Pittsburgh pursuing a career as a clinician-educator. ORCiD: https://orcid.org/0000-0002-4115-7288

**Carla Spagnoletti** is a clinician-educator and general internist. She directs the degree granting programs in Medical Education at the University of Pittsburgh’s Institute for Clinical Research Education.

**Benjamin Miller** is a clinician-educator and pediatric hospitalist. He is an associate director of the pediatric residency program at UPMC Children’s Hospital of Pittsburgh and the director of the Paul C. Gaffney Division of Pediatric Hospital Medicine. ORCiD: https://orcid.org/0000-0002-7354-8056

## Declarations

Dr. Marie Norman has received honoraria in the past for presenting for Magna Online Seminars. Otherwise, the authors have no conflicts of interest or potential declarations of interest to disclose.

## References

[ref1] Agarwal S. and Kaushik J. S. (2020) Student’s Perception of Online Learning during COVID Pandemic. Indian Journal of Pediatrics. Springer, p.554. 10.1007/s12098-020-03327-7 PMC7205599 32385779

[ref2] Ambrose S. A. Bridges M. W. Dipietro M. Lovett M. C. (2010) How learning works: Seven research-based principles for smart teaching. San Francisco: Jossey-Bass.

[ref3] Anderson A. N. (2020) To salvage the semester, college professors make a rapid turn to teaching online. The Washington Post.

[ref4] Bao W. (2020) COVID ‐19 and online teaching in higher education: A case study of Peking University. Human Behavior and Emerging Technologies. 2(2), pp.113–115. 10.1002/hbe2.191 32510042 PMC7262082

[ref5] Boettcher J. V. and Conrad R.-M. (2010) The Online Teaching Survival Guide: Simple and Practical Pedagogical Tips. Jossey-Bass. 15(2), p.322.

[ref6] Borgemenke A. J. Holt W. C. and Fish W. W. (2013) Universal Course Shell Template Design and Implementation To Enhance Student Outcomes in Online Coursework. Quarterly Review of Distance Education. 14(1), pp.17–23.

[ref7] Branswell H. (2020) Americans are underestimating duration of coronavirus crisis, experts say, Stat News. Available at: https://www.statnews.com/2020/04/03/americans-are-underestimating-how-long-coronavirus-disruptions-will-last-health-experts-say/( Accessed: 5 October 2020).

[ref8] Brooks D. C. and Grajek S. (2020) Faculty Readiness to Begin Fully Remote Teaching, Educause Review. Available at: https://er.educause.edu/blogs/2020/3/faculty-readiness-to-begin-fully-remote-teaching( Accessed: 5 October 2020).

[ref9] Brown P. C. Roediger H. L. I. and McDaniel M. A. (2014) Make It Stick: The science of successful learning. Harvard University Press.

[ref10] Chick R. C. Clifton G. T. Peace K. M. Propper B. W. (2020a) Using Technology to Maintain the Education of Residents During the COVID-19 Pandemic. Journal of Surgical Education. Elsevier Inc.,77(4), pp.729–732. 10.1016/j.jsurg.2020.03.018 32253133 PMC7270491

[ref11] Codispoti C. D. Bandi S. Moy J. N. and Mahdavinia M. (2020) Running a virtual allergy division and training program in the time of COVID-19 pandemic. Journal of Allergy and Clinical Immunology. Mosby Inc., pp.1357–1359. 10.1016/j.jaci.2020.03.018 PMC7201123 32243877

[ref12] Darby F. and Lang J. M. (2019) Small Teaching Online: Applying Learning Science in Online Classes. Jossey-Bass.

[ref13] Dedeilia A. Sotiropoulos M. G. Hanrahan J. G. Janga D. (2020) Medical and surgical education challenges and innovations in the COVID-19 era: A systematic review. In Vivo. International Institute of Anticancer Research.pp.1603–1611. 10.21873/invivo.11950 PMC8378024 32503818

[ref14] Dykman C. A. and Davis C. K. (2005) Online Education Forum : Part Two - Teaching Online Versus Teaching Conventionally. Journal of Information Systems Education. 19(2), pp.157–165.

[ref15] EDUCAUSE (2013) EDUCAUSE Homepage | EDUCAUSE.edu. Available at: https://www.educause.edu/(Accessed: 5 October 2020).

[ref16] Ferrel M. N. and Ryan J. J. (2020) The Impact of COVID-19 on Medical Education. Cureus. Cureus, Inc. 10.7759/cureus.7492 PMC7193226 32368424

[ref17] Gewin V. (2020) Five tips for moving teaching online as COVID-19 takes hold. Nature. NLM (Medline). 10.1038/d41586-020-00896-7 32210377

[ref18] Hampton D. Culp-Roche A. Hensley A. Wilson J. (2020) Self-efficacy and Satisfaction With Teaching in Online Courses. Nurse Educator. Ovid Technologies (Wolters Kluwer Health), Publish Ah. 10.1097/nne.0000000000000805 31972846

[ref19] Hofmann H. Harding C. Youm J. and Wiechmann W. (2020) Virtual Bedside Teaching Rounds on Patients With COVID-19. Medical Education. Blackwell Publishing Ltd,54(10), pp.959–960. 10.1111/medu.14223 32403185 PMC7273015

[ref20] Kamenetz A. (2020) “Panic-gogy”: Teaching online classes during the coronavirus pandemic. NPR. 19 March.

[ref21] Kondziolka D. Couldwell W. T. and Rutka J. T. (2020) On pandemics: The impact of covid-19 on the practice of neurosurgery. Journal of Neurosurgery. American Association of Neurological Surgeons, pp.1–2. 10.3171/2020.3.JNS201007 PMC7161161 32276259

[ref22] McMurtrie B. (2020) Students Without Laptops, Instructors Without Internet: How Struggling Colleges Move Online During Covid-19. Chronicle of Higher Education. 66(27), p. N.PAG-N.PAG.

[ref23] Mohr S. C. and Shelton K. (2017) Best practices framework for online faculty professional development: A Delphi study. Online Learning Journal. 21(4), pp.123–140. 10.24059/olj.v21i4.1273

[ref24] Muflih S. Abuhammad S. Karasneh R. Al-Azzam S. (2020) Online Education for Undergraduate Health Professional Education during the COVID-19 Pandemic: Attitudes, Barriers, and Ethical Issues. Research Square. Res Sq. 10.21203/rs.3.rs-42336/v1

[ref25] Norman M. K. (2017) Twelve tips for reducing production time and increasing long-term usability of instructional video. Medical Teacher. Taylor and Francis Ltd,39(8), pp.808–812. 10.1080/0142159X.2017.1322190 28485662 PMC6262844

[ref26] OER Commons (2020). Available at: https://www.oercommons.org/(Accessed: 5 September 2020).

[ref27] OLC Quality Scorecard (2018). Available at: https://onlinelearningconsortium.org/consult/oscqr-course-design-review/(Accessed: 5 August 2020).

[ref28] Pandemic Pedagogy for Visual Arts Professors (2020). Available at: https://www.facebook.com/groups/486971498643714/(Accessed: 5 October 2020).

[ref29] Parisi M. C. R. Frutuoso L. Benevides S. S. N. Barreira N. H. M. (2020) The challenges and benefits of online teaching about diabetes during the COVID-19 pandemic. Diabetes and Metabolic Syndrome: Clinical Research and Reviews. Elsevier Ltd, pp.575–576. 10.1016/j.dsx.2020.04.043 PMC7211751 32413822

[ref30] QM Higher Education Rubric, Sixth Edition , (2018) MarylandOnline, Inc. Used under license. All rights reserved. Retrieved from MyQM.

[ref31] Razavi L. (2020) Students like the flexibility’: why online universities are here to stay, The Guardian. Available at: https://www.theguardian.com/education/2020/may/27/students-like-the-flexibility-why-online-universities-are-here-to-stay(Accessed: 5 October 2020).

[ref32] Reyna J. (2020) Twelve Tips for COVID-19 friendly learning design in medical education. MedEdPublish. Association for Medical Education in Europe (AMEE),9(1). 10.15694/mep.2020.000103.1 PMC10712607 38090049

[ref33] Rose S. (2020) Medical Student Education in the Time of COVID-19. JAMA - Journal of the American Medical Association. NLM (Medline),323(21), pp.2131–2132. 10.1001/jama.2020.5227 32232420

[ref34] Sandhu P. and de Wolf M. (2020) The impact of COVID-19 on the undergraduate medical curriculum. Medical Education Online. Taylor and Francis Ltd. 10.1080/10872981.2020.1764740 PMC7269089 32400298

[ref35] Schmidt S. W. Tschida, Christina M. and Hodge E. M. (2016) How Faculty Learn to Teach Online: What Administrators Need to Know. Distance Education Report. 20(9), pp.1–3.

[ref36] STEM faculty blundering through remote teaching in a pandemic (2020). Available at: https://www.facebook.com/groups/134847691291662/(Accessed: 5 October 2020).

[ref37] Tomlinson S. B. Hendricks B. K. and Cohen-Gadol A. A. (2020) Innovations in neurosurgical education during the COVID-19 pandemic: Is it time to reexamine our neurosurgical training models? Journal of Neurosurgery. American Association of Neurological Surgeons, pp.14–15. 10.3171/2020.4.JNS201012 PMC7164321 32302991

[ref38] Vaill A. L. and Testori P. A. (2012) Orientation, mentoring and ongoing support: A three-tiered approach to online faculty development. Journal of Asynchronous Learning Network. 16(2), pp.111–119. 10.24059/olj.v16i2.256

[ref39] Wayne D. B. Green M. and Neilson E. G. (2020) Medical education in the time of COVID-19. Science Advances. NLM (Medline),6(31), p.eabc7110. 10.1126/sciadv.abc7110 32789183 PMC7399480

[ref40] Weiner S. (2020) No classrooms, no clinics: Medical education during a pandemic, AAMC. Available at: https://www.aamc.org/news-insights/no-classrooms-no-clinics-medical-education-during-pandemic( Accessed: 14 May 2020).

[ref41] Welcome to the One Button Studio (2020) Penn State Teaching and Learning with Technology. Available at: https://onebutton.psu.edu/( Accessed: 5 October 2020).

[ref42] Wolanskyj-spinner A. P. (2020) COVID-19 : The Global Disrupter of Medical Education. American Society of Hematology Clinical News.pp.1–4.

[ref43] Zhou T. Huang S. Cheng J. and Xiao Y. (2020) The distance teaching practice of combined mode of massive open online course micro-video for interns in emergency department during the COVID-19 epidemic period. Telemedicine and e-Health. Mary Ann Liebert Inc. 26(5), pp.584–588. 10.1089/tmj.2020.0079 32271650

