# Supplementary material for: Twelve Tips for Medical Education Leadership to Help Faculty in the Transition to Online Teaching
Source: MedEdPublish (2016). 2020 Dec 9;9:275. [Version 1] doi: 10.15694/mep.2020.000275.1 (PMC10697632; doi:10.15694/mep.2020.000275.1)
Supplement: Peer review history

# Reviewer report: Reviewer response for version 1

**DOI:** 10.21956/mep.20202.r31272

**Published:** 2020-12-12

**Authors:** Samar Aboulsoud

**Affiliations:** 1 CAIRO UNIVERSITY, SCHOOL OF MEDICINE

This review has been migrated. The reviewer awarded 5 stars out of 5 Dear Authors,Thank you for giving me the opportunity to review this interesting article. I strongly agree with you on the importance of the topic and the themes you included in the paper. In my opinion, one of the “positive” effects of the COVID 19 era is that it stimulates innovational thinking and creative solutions in order to address current challenges and issues that are not essentially handled by traditional methods.I think you nicely summarized and listed the important supportive elements and skills that can ensure an effective transition to virtual learning. I believe this is a door opener for further research in other e-learning related domains e.g., the effect of cultural variations on e-learning, addressing e-learning challenges in resource restricted environments and e-learning management throughout the medical education continuum (UG,PG,CPD).One final thought; while we mostly focus on the technical aspects of e-learning, however, I believe that it is crucial to reinforce its educational aspects and models. In other words, it is imperative to ensure that faculty members are capable of transferring the full classroom learning experience to a virtual learning space that is effective and measurable. This includes, but is not limited to, mastering interactive small and large group teaching and discussions; secure& reliable assessment methods and virtual mentorship.Thank you again for a great article.

Reviewer Expertise:

NA

No decision status is available

# Reviewer report: Reviewer response for version 1

**DOI:** 10.21956/mep.20202.r31269

**Published:** 2020-12-12

**Authors:** Mary Higgins

**Affiliations:** 1 University College Dublin, National Maternity Hospital

This review has been migrated. The reviewer awarded 4 stars out of 5 Thank you for this very practical and well referenced paper. I cannot agree more with Subha about the concept of cognitive overload for clinician educators, and while the idea of offering books for reading is laudable, I think what would be preferable would be to have a technician who can help. That, or the ABC of technology enhanced/provided learning. I would have also been grateful to have some tips on how to use technology to return students to more clinical placements, as at some point this will need to happen. Who better to learn from but from the patient themselves, in this situation using technology?

Reviewer Expertise:

NA

No decision status is available

# Reviewer report: Reviewer response for version 1

**DOI:** 10.21956/mep.20202.r31271

**Published:** 2020-12-10

**Authors:** Helena Filipe

**Affiliations:** 1 Hospital of the Armed Forces/PL-EMGFA

This review has been migrated. The reviewer awarded 4 stars out of 5 Thank you for the opportunity to review this useful and interesting paper.Distance as a “new” safety measure has been a ubiquitous word since the pandemic started. I would choose to say physical distance instead of social distance as we always have devised a way including now, to feed our social nature. Lately synchronous social interaction became more than ever one of the most effective means to get in touch within our communities.I appreciated the emphasis on the critical need for medical education departments’ leadership to support a consistent and robust online learning plan. Completely agree when the Authors underline the thought of continuity of such development and implementation efforts to include the experience in future programs. Inasmuch, the pandemic may well imprint a longer lasting need for a sustained elearning methodology or at least the need for hybrid models.Authors provide us with good guidelines full of pragmatic measures to implement and draw our attention to the importance of adaptation of onlineprograms in accordance with the learning environment culture and resources, faculty and learner specific needs.For instance, providing coached training opportunities for faculty to try online tools and platforms looks thoughtful, feasible and allowing for much better outcomes.Appreciated tip 10 mentioning the importance of networking with educators experienced with online teaching and providing faculty with hands-on training opportunities on online tools and resources. Already widely used for personal and practical purposes, friendly-user social networks can be adapted for medical education as local facebook groups may be an example. These can work to straighten personal and professional members’ bonds and allow for developing new learning in a collaborative manner. We are happy to share our insight on Filipe HP, Mack HG. Teaching through social media. Ann Eye Sci 2019; 4:28.Either face to face or online, learning experiences will be better if unfolding in a supportive and trustful environment where the learning group, the learning community members can feel openness to share their views, experience, concerns and aspirations, learn and grow. The learning community will progress with a constructive impact in the organization. The importance of planning and constant improving by piloting and iteration is focused.The updated bibliography will be very helpful to the medical education community in general. Would like to mention a related recent paper published in MedEdPublish: Mealey E, 2019, 'Developing an online continuing professional development course for busy healthcare professionals: 12 tips for course developers', MedEdPublish, 8, [3], 21Thank you for sharing your experience and insight forward in your paper.

Reviewer Expertise:

NA

No decision status is available

# Reviewer report: Reviewer response for version 1

**DOI:** 10.21956/mep.20202.r31270

**Published:** 2020-12-09

**Authors:** Subha Ramani

**Affiliations:** 1 Harvard Medical School, Brigham and Women's Hospital

This review has been migrated. The reviewer awarded 4 stars out of 5 The ongoing pandemic has made online teaching and learning mandatory. I agree with the authors that this mode of education is likely to persist beyond the pandemic. Despite the challenges that have forced the change, educators, institutions and organizations have discovered the benefits of virtual education and the lessons learned will be very relevant in the future. For eg, there are amazing educational conferences organized all around the world and it is impossible for faculty or learners to just travel to several to attend in person. The virtual environment has widened access, facilitated global conversations in a way that was not possible in the past, stimulated cross cultural networking and collaboration.The 12 tips paper is well written, well referenced and quite concise and to the point. I agree with the tips that have been emphasized.My one concern is adding to the burden of faculty whose education provided them expertise in their discipline and are not being asked to also be technology experts. The cognitive load is real and could impair their well being.I would recommend that institutional leaders do their due diligence by hiring educational technologists to help the teacher design online curricula, manage the LMS and also have staff with technology expertise ready to help during synchronous sessions.Another aspect of virtual teaching and learning is the physical space where the teaching is done. Institutions could have experts in space design advise and even provide equipment that would be ergonomic for those with a large online teaching burden. The physical effects of long hours of virtual teaching are only beginning to be described and these are real.All health professions educational leaders could find this article useful

Reviewer Expertise:

NA

No decision status is available
